# Detection of Left Ventricular Regional Function in Asymptomatic Children with beta-Thalassemia Major by Longitudinal Strain and Strain Rate Imaging

**DOI:** 10.4274/Tjh.2012.0065

**Published:** 2013-09-05

**Authors:** Ali Bay, Osman Başpınar, Göksel Leblebisatan, Ali Seçkin Yalçın, Ahmet İrdem

**Affiliations:** 1 Gaziantep University Medical Faculty, Department of Pediatric Hematology, Gaziantep, Turkey; 2 Gaziantep University Medical Faculty, Department of Pediatric Cardiology, Gaziantep, Turkey; 3 Gaziantep Children’s Hospital, Department of Pediatric Hematology, Gaziantep, Turkey

**Keywords:** childhood, Regional left ventricular cardiac function, Strain, Strain rate imaging, Thalassemia

## Abstract

**Objective:** Cardiac failure due to iron overload remains the most common cause of death in patients with beta-thalassemia major. This study aimed to evaluate myocardial function in children with beta-thalassemia major using standard echocardiography technique and strain rate imaging.

**Materials and Methods:** Conventional echocardiographic analysis, tissue velocity imaging, and strain/strain rate imaging of the left ventricle were evaluated in 48 children with beta-thalassemia major (19 girls, 29 boys; 8.39±4.05 years) and 22 healthy children (11 girls, 11 boys; 8±3.72 years).

**Results:** Conventional echocardiographic examinations revealed that beta-thalassemia patients had larger left ventricular end-systolic diameter, end-diastolic and end-systolic volume, left ventricular mass index, and mitral early/late diastolic flow velocity ratio (p<0.05). Strain and strain rate imaging study of the basal lateral wall of the left ventricle was higher in patients than in controls, at p=0.035 and p=0.008, respectively.

**Conclusion:** We found that superior systolic strain and strain rate imaging of the left ventricle indicated the presence of regional systolic function in the left ventricular wall. We suggest that left ventricle volume and mass index parameters might be more sensitive than the other conventional and strain/strain rate imaging parameters during childhood. However, the adulthood strain and strain rate imaging values may be lower than controls, exceeding the critical level of iron overload.

**Conflict of interest:**None declared.

## Abstract (tr)

**Amaç:** Beta talasemi majörlü hastalarda demir yüklenmesine bağlı kalp yetmezliği en sık ölüm nedenidir. Bu çalışmada beta talasemi majörlü çocuklarda standart ekokardiyografi ve strain rate görüntüleme ile myokardiyal fonksiyonun değerlendirilmesi amaçlanmıştır.

**Gereç ve Yöntemler:** Çalışmamızda rutin ekokardiyografik değerlendirme ile beraber sol ventrikülün doku velosite görüntülenmesi, strain/strain rate görüntülemesi beta talasemi majörlü 48 çocukta (19 kız, 29 erkek; 8.39±4.05 yıl) ve 22 sağlıklı çocukta (11 kız, 11 erkek; 8±3.72 yıl) ölçülmüştür.

**Bulgular: **Rutin ekokardiyografik değerlendirmede beta talasemi majörlü hastalarda daha geniş sol ventrikül sistol sonu çap, diyastol ve sistol sonu hacmi olduğu, kitlesinin artmış olduğu ve mitral kapak erken/geç diyastolik akım oranının artmış olduğu görüldü (p<0.05). Sol ventrikül bazal lateral duvarı strain ve strain rate görüntülemesi değerleri hasta grubunda kontrole göre anlamlı yüksek değerdeydi (sırası ile p=0.035 ve p=0.008).

**Sonuç:** Sol ventrikül superior strain ve strain rate görüntülemesinde sol ventrikül duvarının bölgesel sistolik fonksiyonunu koruduğunu bulduk. Çocukluk çağında sol ventrikül hacim ve kitle indeksi göstergelerinin diğer geleneksel ve strain/strain rate görüntüleme göstergelerine göre daha duyarlı olabileceğini düşünmekteyiz. Bununla beraber, erişkin döneminde demir yüklenmesinin kritik seviyeyi aşması ile strain ve strain rate görüntüleme değerleri kontrol olgularınınkinden daha düşük bulunabilir.

## INTRODUCTION

Cardiac failure due to iron overload remains the most common cause of death in patients with beta-thalassemia major, accounting for up to 71% of all deaths from this disease [1,2]. Cardiac complications are related to left ventricle dysfunction leading to gradual cardiac failure and cardiogenic death. Standard echocardiographic measurements may remain normal until late stages during this disease process.

A number of cardiological parameters of left ventricular (LV) function have therefore been assessed to determine their efficacy in identifying early myocardial iron overload in order to prevent heart failure and avert its poor prognosis through increased chelation therapy. The stored iron in the heart is heterogeneous. Regional myocardial asynchrony characterizes diastolic abnormalities even in the absence of changes in systolic functions reported in thalassemia patients and, thus, LV diastolic function may be more sensitive as an early marker of myocardial iron overload. A number of techniques used in clinical practice have been utilized to assess diastolic function in thalassemia major [3,4,5].

Strain rate imaging (SRI) is a new noninvasive echocardiographic method for the analysis of local myocardial stress. SRI is potentially superior to tissue Doppler imaging (TDI) in regional myocardial function assessment [6,7,8]. This study aimed to evaluate myocardial function in children with beta-thalassemia major by using the standard echocardiography technique TDI and SRI and to compare them with healthy controls. 

## MATERIALS AND METHODS

**Patient Population **

Two distinct groups were studied. The patient group comprised 48 beta-thalassemia major patients (19 girls, 29 boys; median 8, mean 8.39±4.05 years). Thalassemia major patients were selected from cases in follow-up in the Gaziantep University Pediatric Hematology Department and Gaziantep Children’s Hospital. The diagnosis of thalassemia was based on hemogram, blood smear, hemoglobin electrophoresis, and clinical evaluation. All patients were under chelation therapy with an oral iron chelator (deferasirox, 30 mg/kg/d) or a parenteral iron chelator (deferoxamine, 40 mg/kg 5 days a week). Serum ferritin levels were noted as ng/mL. Inclusion criteria were diagnosis of beta-thalassemia major, normal renal function, normal left ventricular functions, normal estimated pulmonary pressures by echocardiographic Doppler evaluation, and absence of congenital or acquired structural heart or lung diseases. All thalassemia patients had asymptomatic heart failure and were in New York Heart Association functional class I. They had been receiving blood transfusions since the age of 6 months to 2 years. Twenty-three (47.9%) of the patients had been splenectomized. Severity of iron overload was defined by serum ferritin level. Twenty-two healthy children (11 girls, 11 boys; 8±3.72 years) without history of cardiac disease were included in the study as a control group. All children were in normal sinus rhythm and had normal resting 12-lead electrocardiographs. All studies were performed in accordance with the rules of the local ethics committee. Informed consent was obtained from all participants prior to the study.

**Echocardiography**

Conventional echocardiography (Vivid 3, GE Vingmed Ultrasound, Horten, Norway) and strain and strain rate imaging were performed by the same experienced pediatric cardiologist. Echocardiographic images were obtained in the parasternal long-axis and short-axis, and apical 2-chamber and 4-chamber views were obtained with standard transducer positions. Conventional echocardiographic measurements were done according to the American Society of Echocardiography guidelines [9]. Left ventricle mass was calculated using the Devereux formula [10]. Left ventricle mass index was calculated by dividing the left ventricle mass by body surface area. All examinations were videotaped and contemporary electrocardiography traces were recorded. From the parasternal long-axis view of the LV end-diastolic and end-systolic diameters, interventricular septal and posterior wall thicknesses were expressed in millimeters. We measured LV end-systolic and end-diastolic (EDV) volumes from the apical 4-chamber view. Left ventricle fractional shortening (FS) and ejection fraction (EF) were measured using the Teichholz formula. LV filling was evaluated by pulse wave Doppler from the apical 4-chamber view with the sample volume position at the tips of the mitral valve, and velocities in early (E) and late (A) diastole were recorded, in addition to the calculation of the E/A ratio. Furthermore, myocardial velocities of the left ventricle were evaluated by tissue velocity imaging (TVI), strain (S), and SRI.

Digital data of color TDI were transferred for off-line analysis with EchoPAC-PC software (GE Vingmed Ultrasound). Scanning was performed longitudinally from the apex to acquire apical 2-chamber and 4-chamber views with a 3-MHz transducer and a frame rate of 100 ± 20 frames/s, depending on the heart rate, to minimize the noise level. Myocardial velocities were measured for the local motion of a tissue by TVI and S, and the local rates of deformations were measured by SRI. Longitudinal strain and strain rate in the basal septal, basal lateral, mid-septal, and mid-lateral wall were estimated by measuring the spatial velocity gradient over a computational area of 3 mm × 5 mm. Tissue velocity, strain, and strain rate were obtained at each site from 3 consecutive b eats and average values were calculated. End-diastole was defined as the R peak in echocardiography, and end-systole was defined as the end of the T wave in echocardiography. Peak positive systolic values were calculated from the extracted curve. From the 4-chamber view, the TVI sample volume was located sequentially at septal and lateral sites of the valvular ring. Mean values of early diastolic, late diastolic, and systolic myocardial velocities were calculated as cm/s.

**Statistical Analysis**

Statistical analysis was performed using SPSS 11.0 (SPSS Inc., Chicago, IL, USA). All data were expressed as mean ± standard derivation. Comparison of measurements between 2 groups was analyzed with the unpaired Student t-test. Correlation coefficients between various measurements were determined by the linear regression analysis. A p value of less than 0.05 was considered significantly different. 

## RESULTS

The demographic characteristics of the patients with beta-thalassemia major and the control group are presented in Table 1. The 2 groups were similar regarding age and body surface area. Conventional echocardiographic examinations revealed that beta-thalassemia patients had larger LV end-systolic diameter (cm), end-diastolic and end-systolic volume (mL), LV mass index (g/m2), and mitral early/late diastolic flow velocity ratio. Mitral early-to-late diastolic flow velocity ratio as determined by conventional echocardiography was significantly higher in thalassemia major patients compared to the control group (Table 2).

In the TVI technique, left ventricle lateral late diastolic myocardial velocity was lower in beta-thalassemia patients compared to the control group, and the lateral early-to-late diastolic myocardial velocity ratio was higher in beta-thalassemia patients compared to the control group (Table 3). Strain and SRI study included the following measurements: basal lateral and septal segments and mid-lateral and septal segments of the LV walls. The basal lateral walls’ S and SRI measurements were higher in patients compared to controls: p=0.035 and p=0.008, respectively (Tables 4 and 5).

Pearson’s correlation coefficient showed significant correlations between the serum ferritin levels and the following echocardiographic variables: LV end-diastolic volumes (r=0.288, p=0.049), LV end-systolic volumes 

(r=0.354, p=0.015), EDV (r=0.288, p=0.049), EF (r=-0.380, p=0.008), FS (r=-0.378, p=0.009), E (r=-0.302, p=0.044), septal early TVI (r=-0.329, p=0.026), and lateral systolic TVI (r=-0.330, p=0.025). 

## DISCUSSION

The most common cause of morbidity and mortality in thalassemia major patients is cardiomyopathy due to iron overload. Heart disease is mainly expressed by a particular cardiomyopathy that progressively leads to heart failure and death [1,2,11]. Iron toxicity has been attributed to the production of free oxygen radicals, as a result of the Fenton and Haber-Weiss reactions, which take place in the presence of free iron, the most toxic form of iron. 

Since cardiac function remains normal until late stages in the spectrum of iron cardiomyopathy, other tools are necessary to anticipate and prevent iron cardiomyopathy. Cardiac magnetic resonance imaging, tissue Doppler echocardiography, radionuclide angiography, and stress echocardiography are useful imaging studies for the detection of early cardiac dysfunction. Recently, tissue Doppler echocardiography and radionuclide angiography (with exercise or low-dose dobutamine stimulation) have been shown to detect regional wall motion abnormality, even in early-stage thalassemic patients [11]. This finding may also reflect patchy, nonhomogeneous deposition of iron in cardiac muscle.

Fitchett et al. demonstrated the deposition of iron within the myocytes rather than the interstitium, and this cardiac iron deposition was patchy [12]. Lattanzi et al. and Vogel et al. showed that regional wall motion abnormalities and regional iron overload were seen in patients with beta-thalassemia major [13,14]. These regional changes can be easily detected with an echocardiographic assessment including TVI and S/SRI analysis.

The novel contribution of our study is the demonstration of the superiority of SI over the conventional echocardiographic parameters, LVEF and LVFS, in the detection of regional myocardial function. Our patients had superior systolic S and SRI of the LV lateral wall, indicating the presence of regional systolic function in the left ventricular wall. We suggest that LV volume and mass index parameters might be more sensitive than the other conventional and S/SRI echocardiographic parameters. However, adulthood S/SRI sensitivity may be lower than in the controls.

Regular clinical follow-up is strongly recommended for precocious detection of symptoms and signs of myocardial dysfunction [15]. On the hypothesis that longitudinal ventricular function may give early information about impending ventricular function, we used TVI and S/SRI measurements [6,7]. Regional findings suggest the presence of differences in myocardial function between the asymptomatic patients with beta-thalassemia major in the early stage of disease and the healthy controls. These regional changes can be easily detected with an echocardiographic assessment including TVI and S/SRI analysis. 

Previous studies have shown that beta-thalassemia major is frequently associated with progressive LV dysfunction, leading to congestive heart failure. LV diastolic function, measured by traditional measurements such as transmitral flow recordings, is preserved until the final stages of diseases [16,17,18]. Conventional Doppler indices frequently lead to incorrect diagnosis. 

In our patients, chronic iron overload resulted in significant increase in serum ferritin and significantly increased LVMI. However, EF and FS were not altered significantly in patients compared with controls, suggesting preserved systolic functioning until late stages of the disease. This finding is consistent with those of Parale et al. and Seliem et al. [18,19].

Magri et al. suggested TDI and strain imaging as a potential method for detecting early stages of abnormal iron deposition [20]. Olivieri et al. and Bosi et al. demonstrated that serum levels of ferritin below 2500 ng/mL were considered as the safe level [1,21]. Our mean value of serum ferritin was at this level. Fitchett et al. demonstrated iron deposition within the myocytes rather than the interstitium and this cardiac iron deposition was patchy [12]. Lattanzi et al. and Vogel et al. showed that regional wall motion abnormalities and regional iron overload were seen in patients with beta-thalassemia major [13,14]. We found wall motion abnormality located in the basal lateral segment of the LV wall. There is no clear explanation for this observation. 

S/SRI offers a new way of measuring regional tissue deformation noninvasively with both good spatial and temporal resolution. Authors have demonstrated it to be a tool that would appear to offer reproducible and reliable data [8]. Our findings suggest that S/SRI parameters have the potential to detect early myocardial changes that precede abnormal LV filling. 

This study demonstrates the ability of S/SRI to accurately depict regional myocardial function in asymptomatic beta-thalassemia patients with normal systolic and diastolic function. LV dilatation or increased LVMI was balanced with increased basal segment S/SRI measurements. During this period, increased LV dilatation may progress to lower S/SRI values and to LV diastolic and systolic function disorders. Until the critical level of iron overload, peak systolic strain and SRI may accurately reflect local systolic function, and we may say that iron overload is not related to a specific area of LV myocardium and that cardiac changes are possibly related to a threshold level of iron overload. 

While serum ferritin levels remain above 2500 µg/L, echocardiographic measurements such as basal TVI, S, and SRI parameters might show compensatory increase although LV dilatation starts with normal systolic function. This compensation would probably be inadequate at greater ferritin levels and systolic and diastolic functions would globally decrease eventually. Therefore, clinical manifestations usually occur in adulthood rather than childhood. According to the Frank-Starling law, if heart volume increases, this compensation might be inadequate, and these parameters worsen through adulthood. 

Accurate assessments of cardiac dysfunction and cardiac iron status are currently based on imaging techniques. The T2-star magnetic resonance is currently the best noninvasive modality to estimate iron in the heart and other organs [22]. In addition, it can be used for monitoring myocardial iron levels during iron chelation therapy. Unfortunately this examination cannot be performed in our hospital; we could not correlate this technique with the results.

One of the limitations of our study is that we could not compare the patients within every ferritin level. In asymptomatic patients, normal systolic and diastolic functions might be related to good LV compensation. Strengthening the chelation treatment in this period may normalize these measurements. Even when LVEF and LVFS are normal, increased LV mass and volume may be seen. In this study we found compensatory increase in S and SRI measurements, and these parameters would probably worsen first to be followed by cardiac failure appearing due to decreased LVEF and LVFS.

## CONFLICT OF INTEREST STATEMENT

The authors of this paper have no conflicts of interest, including specific financial interests, relationships, and/ or affiliations relevant to the subject matter or materials included.

## Figures and Tables

**Table 1:**
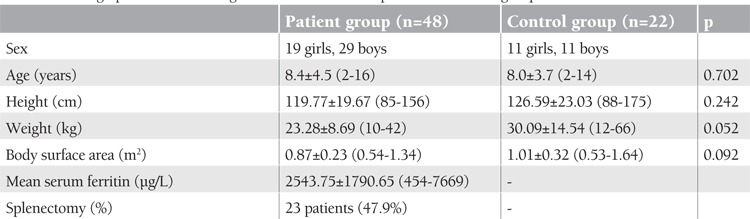
Demographic and hematologic characteristics of the patient and control groups

**Table 2:**
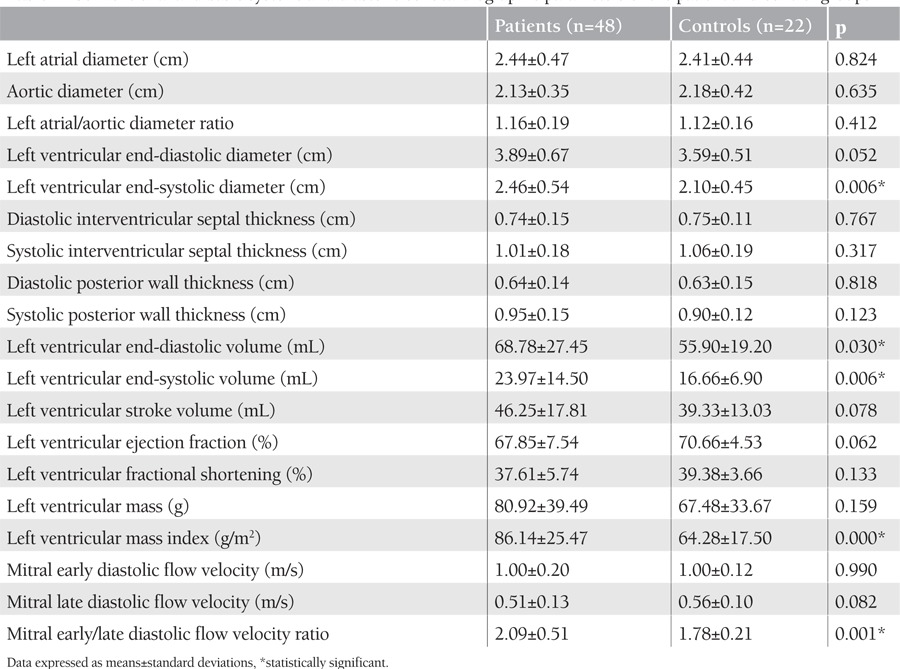
Conventional and basic systolic and diastolic echocardiographic parameters of the patient and control groups

**Table 3:**
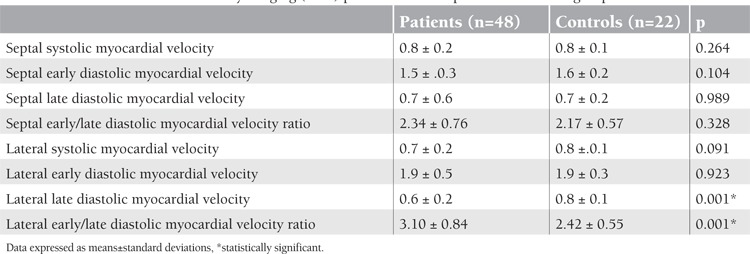
Left ventricular tissue velocity imaging (cm/s) parameters of the patient and control groups

**Table 4:**
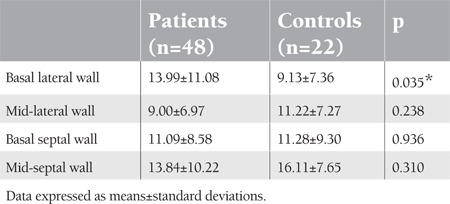
Left ventricular strain (%) parameters of the patient and control groups

**Table 5:**
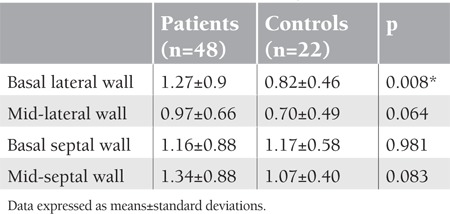
Left ventricular strain rate imaging (1/s) parameters of the patient and control groups
